# TUCAN: A molecular identifier and descriptor applicable to the whole periodic table from hydrogen to oganesson

**DOI:** 10.1186/s13321-022-00640-5

**Published:** 2022-09-28

**Authors:** Jan C. Brammer, Gerd Blanke, Claudia Kellner, Alexander Hoffmann, Sonja Herres-Pawlis, Ulrich Schatzschneider

**Affiliations:** 1 Institut für Anorganische Chemie, RWTH Aachen, Landoltweg 1a, 52074 Aachen, Germany; 2 StructurePendium Technologies GmbH, Reulsbergweg 5, 45257 Essen, Germany; 3 Institut für Anorganische Chemie, Julius-Maximilians-Universität Würzburg, Am Hubland, 97074 Würzburg, Germany

**Keywords:** Cheminformatics, Molecular representation, Chemical identifier, Canonicalization, Molecule isomorphism, Line notations, Molecular graphs, Software library, Python

## Abstract

TUCAN is a canonical serialization format that is independent of domain-specific concepts of structure and bonding. The atomic number is the only chemical feature that is used to derive the TUCAN format. Other than that, the format is solely based on the molecular topology. Validation is reported on a manually curated test set of molecules as well as a library of non-chemical graphs. The serialization procedure generates a canonical “tuple-style” output which is bidirectional, allowing the TUCAN string to serve as both identifier and descriptor. Use of the Python NetworkX graph library facilitated a compact and easily extensible implementation.

**Graphical Abstract:**

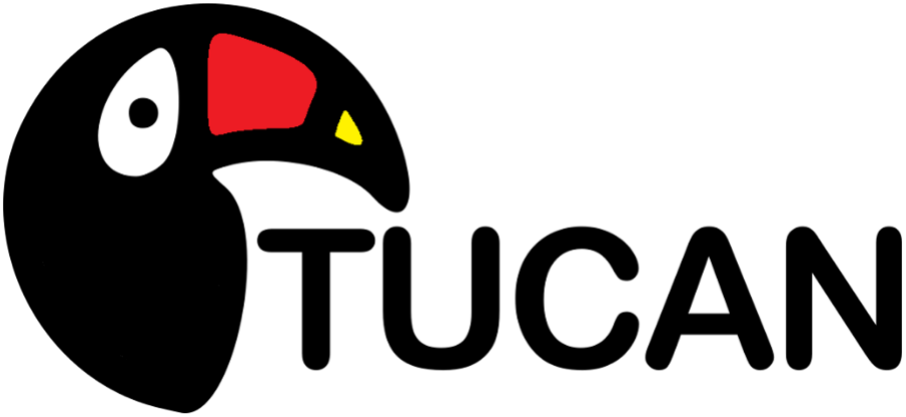

## Background

**Fig. 1:**
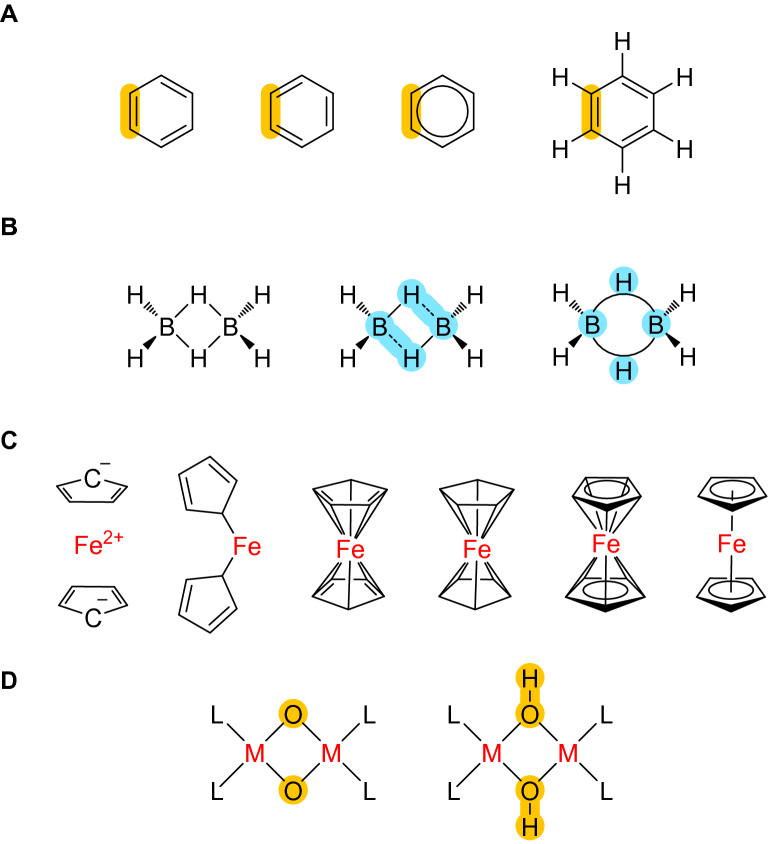
**A** Four different structural representations of benzene (C_6_H_6_) with one C–C bond highlighted in orange to serve as a reference, only the rightmost structure shows explicit hydrogens, **B** three different structural representations of diborane (B_2_H_6_), only the left structure preserves the correct symmetry but has an electron count that is too high by four electrons, the center structure tries to preserve the electron count by using “zero-order” bonds [2] indicated as dashed lines at the expense of an artificially lowered symmetry, and the right structure uses “banana bonds” to capture the three-center-two-electron (3c–2e) nature of the B–H–B bonding which however cannot be represented with traditional graph concepts, **C** different representations of ferrocene ([Fe(C_5_H_5_)_2_]), with only the 3rd to 5th from the left properly representing the 10 equal Fe–C bonds observed in X-ray structures, **D** Without explicit hydrogen atoms, it is impossible to distinguish between the bis-µ-oxo (left) and bis-µ-hydroxo (right) structures often encountered in inorganic chemistry, as there is no known algorithmic way to decide whether H atoms have to be added to the M_2_O_2_ core or not

For several decades, cheminformatics has largely focused on organic chemistry [1], since Lewis structures mostly convey a simple bonding scheme that applies well to this domain of chemistry. Lewis structures entail the simplification that a straight line “–” connecting two element symbols indicates two electrons in a localized two-center-two-electron bond (2c–2e). The lines serve an important role in the “book-keeping” of the electrons assigned to a particular center. Similarly, a “=” symbol indicates four electrons shared by two atoms, two in a σ- and two in a π-orbital, and “≡” symbolizes six electrons shared by two atoms, two in a σ- and four in two orthogonal π-orbitals. Furthermore, hydrogen atoms are usually not shown when attached to carbon centers (Fig. 1A) and such “implicit hydrogens” are only indirectly taken into account via the assumption of a “standard valence”, with the number of C–H bonds assumed to be equal to the difference of a “standard valence” minus the number of bonds (= lines) that extend from the element symbol in question to its immediate neighbors. This approach has served the organic chemistry community well for over a century and continues to facilitate communication about reaction mechanisms and the prediction of properties.

In contrast, the situation is much less clear in molecular inorganic chemistry, as overlap of the *d*-orbitals allows for bond orders higher than three [3] and metal centers can be present in a wide variety of coordination environments, both in terms of the number of bound ligand atoms (= coordination number) and in the geometrical structure they assume in 3D space (= coordination polyhedron), often leading to complicated stereochemistry [4, 5]. Furthermore, (organo)metal compounds are often characterized by a high degree of electron delocalization and multi-centric bonding, which cannot be captured in simple Lewis formulas (Fig. 1B, C), and there is no algorithmic way to determine the number of implicit hydrogen atoms to be assigned to a metal center or the directly adjacent ligand atoms (Fig. 1D).

This leads to nomenclature in which electrons are not assigned to individual bonds anymore but to extended groups of atoms, as for example in the Enemark–Feltham notation for metal-nitrosyl complexes. Here, instead of assigning individual oxidation states to the atoms and bond orders to the bonds in a MNO moiety, only the sum *n* of the electrons in the metal *d* orbitals and the nitrosyl π^*^ orbitals is indicated as {M(NO)_*x*_}^*n*^ [6].

Therefore, to enable a computer to handle chemical structures representing molecules made up of atoms from all across the periodic table, from hydrogen to oganesson, regardless if they can be synthesized or are merely theoretical, molecular identifiers and descriptors have to be able to handle the full scale of structure and bonding situations from all domains of chemistry.

Classical cheminformatics approaches rely on string representations such as SMILES and InChI, to name only the two most important ones. The *Simplified Molecular Input Line Entry System* (SMILES) can, at least in principle, be applied to all elements of the periodic table [7]. Also, explicit handling of hydrogen atoms is possible when these are defined together with the corresponding heavy atoms, as exemplified by the SMILES notations for the dihydrogen molecule and the ammonium cation, which are [H][H] and [NH4+], respectively. However, SMILES reaches its limits when dealing with bond orders higher than three or encountering strongly delocalized bonding situations, since it allows only four bond types: “*Single, double, triple, and aromatic bonds are represented by the symbols –,* =*, #, and :, respectively*” [7]. Furthermore, the approach of representing cyclic structures already runs into difficulties with simple organometallic compounds such as ferrocene ([Fe(*η*^5^–C_5_H_5_)_2_], Fig. 1C). Finally, in the original version, there is no definition of the stereochemistry of a molecule [7] and while later informal publications describe some limited handling of tetrahedral, square-planar, trigonal–bipyramidal, and octahedral geometries as well as configuration around double bonds and allenes [8], this coverage is incomplete, as it lacks for example square-pyramidal and trigonal-prismatic structures, which are also highly relevant to inorganic chemistry, let alone higher coordination numbers [9]. Furthermore, the International Chemical Identifier (InChI) also relies on the classic Lewis picture, but uses an additional “disconnection approach” which cuts all bonds to metals, thereby severely limiting the meaningful description of most molecular inorganic compounds [10], as with the metal–ligand bonds disconnected, inorganic stereochemistry is completely lost.

Alternative approaches have rarely appeared in the literature, and if so, were not widely accepted, as they turned out to be too complicated. One such notation was published in 1995 by Dietz, in which a chemical structure is represented as sets of numbers and symbols, with sets enclosed in “wavy brackets” and lists of properties (*n*-tuples) enclosed in round brackets [11]. The first set is composed of 3-tuples (*v*, *n*, *Z*) which describe the molecules constituent atoms, with *v* being the number of unshared valence electrons (those which are not involved in any bond). *n* is a consecutive index that runs from 1 through *i*, with *i* equal to the number of atoms in a molecule, and *Z* is either the atomic number or the element symbol, which can be used equivalently. The second set then describes the “bonding systems” of a molecule and is composed of the number of electrons that are involved in each of them, and 2-tuples of atom pairs. With simple localized bonding, for example, dihydrogen is described as ({(0,1,H),(0,2,H)},{(2,{{1,2}})}), with the two zeros indicating that each of the hydrogen atoms has no free electron pair available, 1 and 2 being the atomic index numbers, and in the second set the leading 2 indicating the presence of a 2c–2e bond. Interestingly, this also enables the description of delocalized bonds. For example, diborane (B_2_H_6_, Fig. 1B) is encoded as ({(0,1,B),(0,2,B),(0,3,H),(0,4,H),(0,5,H),(0,6,H),(0,7,H),(0,8,H)},{(2,{{1,3}}),(2,{{1,4}}),(2,{{2,7}}),(2,{{2,8}}),(2,{{1,5},{2,5}}), (2,{{1,6},{2,6}})}), but due to the large number of brackets, it is hard to parse, which potentially kept this approach from gaining wider attention, despite the fact that the notation can represent delocalized multi-centric bonding [11]. Later in this article, a streamlined version of this “tuple notation” will be derived, which removes the redundancy of the Dietz notation and is meant as an alternative to the “linear notations” of InChI and SMILES. In a preliminary study on “*Coordination Complexes for InChI*”, an attempt to extend the InChI towards coordination and organometallic systems, Clark introduced the concept of “zero-order bonds”, which represent bonding interactions between metal and ligand atoms without relying on the “standard valence” [12]. However, this approach still relies on bond orders and the proper placement of formal charges, and is therefore not domain-independent.

## Implementation

### Motivation and concept

Based on the considerations above, it was decided to fundamentally re-think how to derive a chemical identifier and descriptor. The following aspects were considered fundamental to the design of the format, TUCAN (for “tuple canonicalization” or from Spanish “*tu* *canonicalización*” = “your canonicalization”):

- **Input format and normalization**
  A molfile v3000 is used as input, as it is widely accepted in cheminformatics now and generated by most popular chemical editors [13]. In particular, compared to the molfile v2000 [14], it is not limited to a maximum of 999 atoms and bonds, and without the fixed field widths of the older format, is easier to parse [15, 16]. The molfile is considered to be valid and only a number of elemental checks is performed on the input as described below. A check of the chemical validity and some normalization might later be added in the form of a “pre-processor”, but is not within the scope of the current work. Also, an implicit-to-explicit hydrogen “converter” might be implemented as part of a pre-processor in the future. Furthermore, consideration of the stereochemistry will not be part of this initial version of the tool but rather the topic of a separate follow-up publication.
- **Internal data structure**
  The molecule is represented as an *undirected, labeled, and connected graph* without multi-edges or loops [17], with the *atoms* as *nodes* (sometimes called *vertices* or *points*) and the *bonds* as *edges* (also called *links* or *lines*). Both nodes and edges can be assigned further properties which can be used during graph canonicalization. The use of a mature graph library, NetworkX, facilitates working with the graph data structure [18].
- **Canonicalization**
  The canonicalization is—as far as possible—based on topological features of the molecule, to avoid any dependence on concepts and models of structure and bonding that only apply to specific domains of chemistry and are not observables in a quantum chemical sense.
- **Serialization**
  The serialization results in a single string which is *bijective*—in the sense that a unique string is generated for each molecule independent of the ordering of the atoms in the molfile, and that this string is not generated for any other molecule. Furthermore, the identifier string aims to be compact and character-efficient, readable by both computers and humans, and preferentially also be bidirectional, thus allowing the reconstruction of a simple molfile (without the xyz coordinates and bond properties) from the string, thus also serving as a compact descriptor of the molecule.

## Implementation

### Input processing and generation of graph data structure

**Fig. 2:**
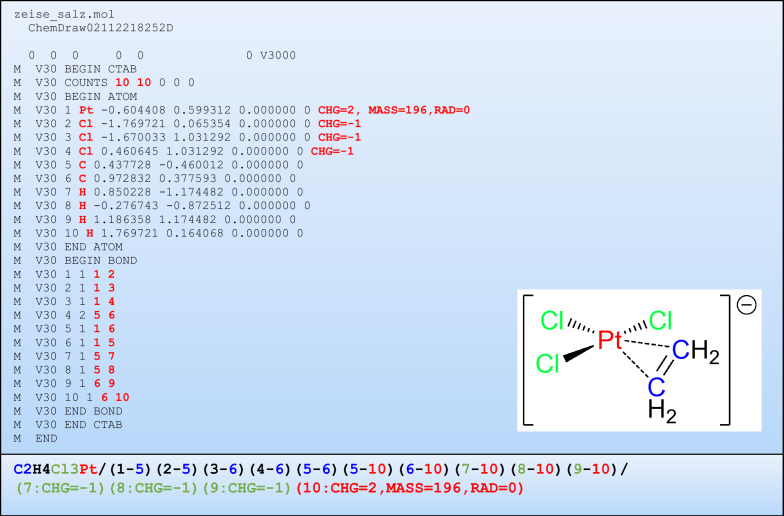
(Top) Molfile in v3000 format for Zeise’s salt (potassium trichloro(ethylene)platinate(II) hydrate, K[PtCl_3_(C_2_H_4_)]⋅H_2_O), with the potassium cation and a lattice water molecule removed for clarity. Generated with ChemDraw 20 using explicit hydrogen atoms. Field values read in by the program are highlighted in bold red font. In the lower right inset, the structure is shown with lines indicating bonding interactions, as defined in the bond block of the molfile. (Bottom) TUCAN string for Zeise’s salt as shown above with color coding of the elements as in the inset

A molfile in v3000 format, which is considered to be valid, serves as the input [14–16]. Three sets of values are extracted from the molfile and used to create a graph (Fig. 2) [15, 16]. First is the number of atoms and bonds from line 6. The second, from the ATOM block for each of the atoms, is the element symbol along with optional CHG, MASS, and RAD fields, which indicate a non-zero atomic charge, a non-standard isotope presence, and the multiplicity, respectively. All further fields, including the (pseudo) 3D coordinates, are discarded, as they are not relevant to TUCAN's approach of explicit hydrogens. The “star pseudo-atom” (“*”), which is used to indicate multi-endpoint bonds required to describe metal π complexes will be supported in future TUCAN versions. The third set of values is extracted from the BOND block.

**Fig. 3:**
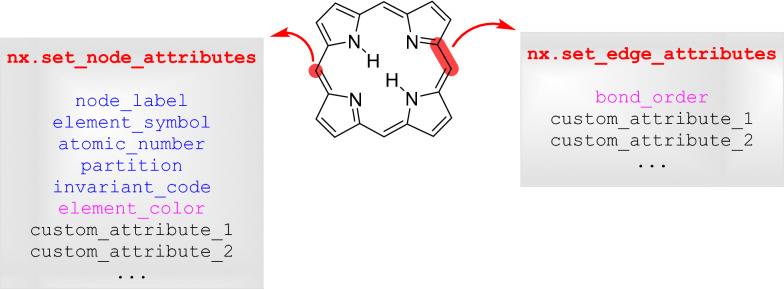
Depiction of the internal data structure: Atoms are represented as nodes and bonds as edges. To the nodes, a number of “node attributes” is attached which are either used in the canonicalization (blue) or in graphical output (magenta). Generally, the edges could also be assigned “edge attributes” but these are not needed for canonicalization. Furthermore, nodes and edges can also be assigned user-defined “custom attributes”, which are also not used in the canonicalization, but might be useful to carry along data, for example for machine-learning applications

Only the atom indices of the two atoms forming a bond are considered, while the bond type is discarded, since it is strongly domain-specific and bond orders beyond four [3], which are nevertheless important to inorganic chemistry, are currently not defined (and will be hard to add to the definition, as bond type values 5 and 6 are already differently assigned in the molfile v3000 format, to types “single or double” and “single or aromatic”, respectively) [15, 16]. An example of a molfile for Zeise’s salt, which it the first organometallic compound that was isolated in pure form [19, 20], is shown in Fig. 2, with the extracted values highlighted in bold red. Importantly, the program handles all hydrogen atoms explicitly, which therefore have to be present in the molfile. Next, the element symbols and bonds are used to create a NetworkX graph data structure [18]. Numerical node labels are assigned to each of the atoms and the element symbol and atomic number stored with each node (Fig. 3C). These are the only chemistry-specific values used throughout the program, which ensures that the canonicalization algorithm is agnostic towards domain-specific concepts of structure and bonding. Then, two additional attributes are assigned to each node. The first is derived from the topology of the molecular graph and includes the partition number and an invariant_code, which is constructed from the atomic number followed by the multiset of neighboring atomic numbers.

The partition number is initialized with a value of zero for each atom (Fig. 3C). The second set of “custom node attributes” is not used in the canonicalization, but can be assigned for other purposes such as the visualization of the molecule. For example, in the current version, the element RGB color as defined in Jmol^1^ is added as an additional node attribute for coloring of the output.

### Canonicalization

**Fig. 4:**
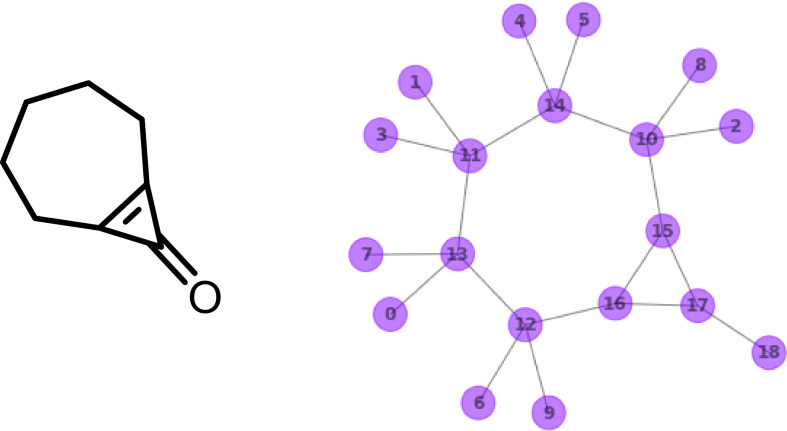
(Left) Lewis formula with implicit hydrogen atoms and (right) molecular structure of bicyclo[5.1.0]oct-1(7)-en-8-one with arbitrary node labels and all nodes (atoms) assigned to the same partition, here highlighted in purple

The canonicalization algorithm employed in this work is based on invariants which were selected to be as independent as possible from domain-specific models of structure and bonding, in contrast to previous approaches [21–23]. Thus, the only invariant which is specific to chemistry is the atomic number, which is derived from the element symbol in the molfile. In the following section, bicyclo[5.1.0]oct-1(7)-en-8-one (C_8_H_10_O) will serve as an example to illustrate the canonicalization (Fig. 4 left) [24].

**Fig. 5:**
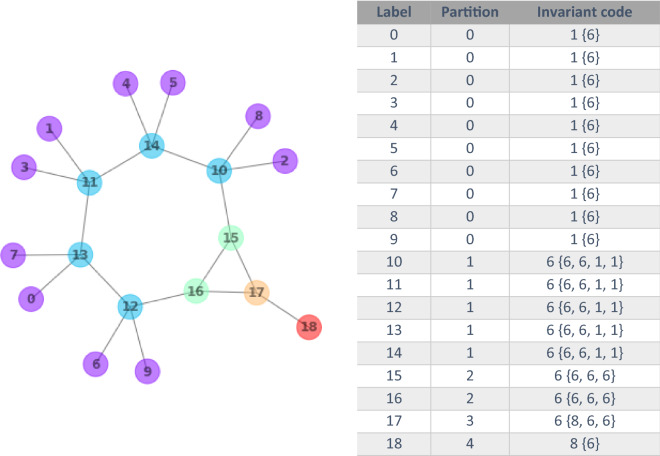
Molecular structure of bicyclo[5.1.0]oct-1(7)-en-8-one with nodes grouped in color-coded partitions 0 through 4 according to their invariant codes

In the first step, the nodes (atoms) are labelled according to the molfile, offset by -1 in order to obtain zero-based labels, shifted if necessary to ensure they are continuous, and assigned to the same partition (Fig. 4 right). Then, an invariant code is constructed for each node, which captures features of the node and its immediate neighbors (those nodes which are connected to the node by a single edge). Specifically, the invariant code consists of a node’s atomic number, followed by the multiset of neighboring atomic numbers, the latter sorted in decreasing order (Fig. 5). In the second step of the canonicalization, all nodes that have identical invariant codes are put in the same partition, such that the 19 atoms of bicyclo[5.1.0]oct-1(7)-en-8-one are placed in five partitions (Fig. 5). For example, nodes 16 and 17 are each connected to three direct neighbors, but the multisets of their neighbors' atomic numbers are different. Node 16 is connected to three carbons, whereas node 17 is connected to two carbons and one oxygen. Similarly, nodes can be discriminated if they belong to the same partition but have a different *number* of direct neighbors. With the multiset of a node’s direct neighbors' atomic numbers, two more invariants are generated: the number of direct neighbors as well as the *configuration* of neighbors.

**Fig. 6:**
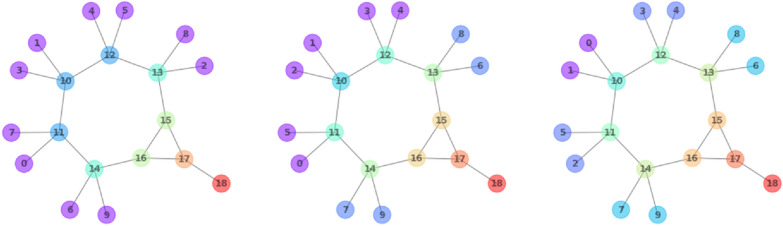
Partitions after first (left), second (center), and final (right) round of refinement

**Fig. 7:**
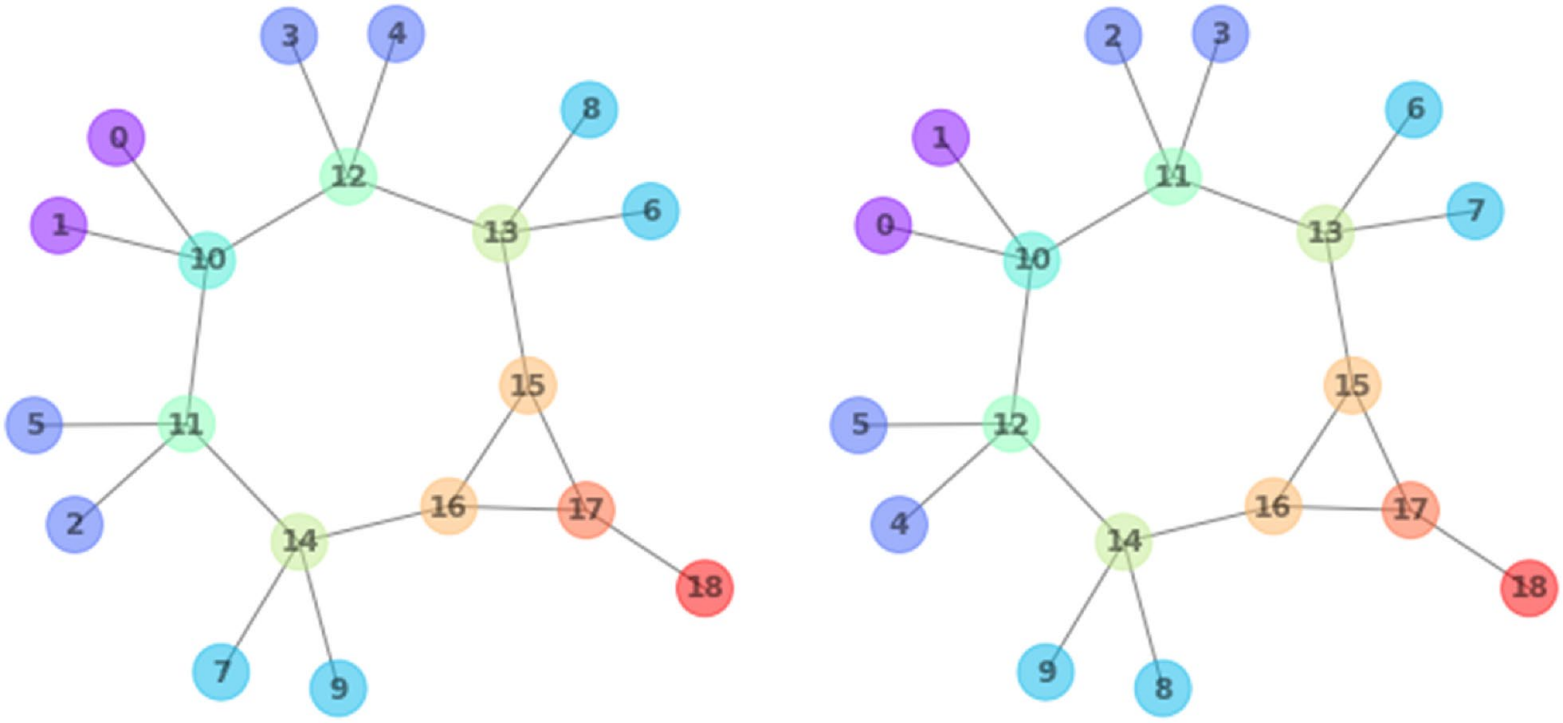
(Left) node labels after the final refinement and (right) after bliss canonicalization

Subsequently, the partitions are iteratively refined with the 1-dimensional Weisfeiler–Lehman algorithm [25, 26], which is known in chemistry as the Morgan algorithm [27]. Two nodes *i* and *j* are assigned to different partitions if they are connected to different multisets of *partitions*. This is repeated iteratively until no node can be assigned to a new partition anymore (Fig. 6). Finally, in order to assign canonical labels to the nodes, the partitioned graph is passed to the bliss algorithm (Fig. 7) [28]. Note that for TUCAN, the canonicalization algorithm is interchangeable, as long as (1) molecules that are compared to each other based on their TUCAN strings are canonicalized with the same algorithm, and (2) the canonicalization algorithm does not use any domain-specific concepts as node invariants, as would be the case for the InChI algorithm for instance.

### Serialization

In the serialization step, the graph data structure is converted into an identifier string, as required for example when searching through large data sets. In addition to the strict requirement for an identifier to be bijective (a unique identifier for each compound and no other compound with the same identifier), it is useful to construct the string in a way which allows automatic reconstruction of the molecular topology and ideally, the representation should also be intuitive enough for humans to reconstruct the molecular graph. Current string identifiers and descriptors such as InChI and SMILES use a “linear chain with branches” approach [29], which requires that one keeps track of the branching points in the main chain and then traverses all of the additional chains, which increases the complexity of the serialization algorithm. The predominance of this approach can be traced back to some of the earliest publications on molecule linearization, for example by Hiz and Eisman [30, 31], but possibly has its roots in “semi-sum formulas” such as CH_3_CH_2_OH for ethanol, that date back as far as the 1860s [32]. Alternative concepts have been proposed for example by Dietz [11], but as discussed in the introduction, their acceptance was impeded by a confusing nomenclature involving many nested brackets. However, the Dietz nomenclature can be simplified, first by removing the atomic index numbers in the first “atom set” under the assumption that the atoms are ordered by increasing atomic number (which is not the case in the original Dietz paper). Then, atomic index numbers can be inferred from the sum formula. For example, for ferrocene (C_10_H_10_Fe) (Fig. 1C), the ten hydrogen atoms will get atomic index numbers of 1 through 10, the ten carbon atoms 11 through 20, and finally the iron center receives the index number 21. As our approach is based on explicit hydrogens, there is no need to keep track of unshared valence electrons and therefore, the complete first set of 3-tuples in the Dietz notation can be replaced by the sum formula in the Hill format—C and H first, followed by the remaining elements in alphabetical order of their element symbols [33]. Furthermore, in the second set used by Dietz to describe the “bonding systems” of a molecule, the number of electrons that are involved in these is also not needed for canonicalization and can be discarded as well, leaving only 2-tuples of atom pairs. These can be obtained from the edge list of the molecule graph. In TUCAN, each 2-tuple is composed of the node labels of two connected atoms [i.e., (label atom_A-label atom_B)]. The numerical labels of atom_A and atom_B are first arranged in increasing order (thus, 2–6 and not 6–2) and then the whole list is sorted by increasing node index numbers of each 2-tuple. Each edge is present only once in the edge list, in contrast for example to an adjacency matrix representation. Combined with the sum formula, this allows inferring which node is associated with which element. Additional information on the nodes, such as the local charge, non-standard isotope distribution, and multiplicity are added as an additional node features list. Different blocks are separated by a slash (“/”) and appear in the following order: (1) sum formula, (2) 2-tuple edge list, (3) node features list, and (4) potential further custom data. The only mandatory block is the sum formula, which has to be present even for monoatomic species as well as compounds in which the individual atoms are all disconnected. For example, the TUCAN string representation for Zeise’s salt (Fig. 2) according to this format is:

C2H4Cl3Pt/(1–5)(2–5)(3–6)(4–6)(5–6)(5–10)(6–10)(7–10)(8–10)(9–10)/(7:CHG=−1)(8:CHG=−1)(9:CHG=−1)(10:CHG=2,MASS=196,RAD = 0)

Here, the sum formula indicates that the molecule consists of two C, four H, three Cl, and one Pt atom. Since increasing labels are assigned to atoms with increasing atomic number, it can be inferred that the labels 1 through 4 pertain to H atoms, labels 5 and 6 to C atoms, labels 7 through 9 indicate the Cl atoms and finally, label 10 pertains to the central Pt atom. Thus, the first four 2-tuples represent the C–H bonds, followed by the central C–C bond of the ethylene ligand (5–6), the two metal–carbon bonds (5–10 and 6–10), and the three metal-chlorido bonds. Finally, the node attributes are also presented as a n-tuple list, in which n is the number of node attributes per node plus one for the node number. Different node attributes are given in FIELD = VALUE format without blanks and separated by commas, if there is more than one node attribute assigned. The node number and the following FIELD = VALUE definitions are separated by a “:” sign. For each node, the node attributes are sorted lexicographically according to the FIELD names and the tuple list is sorted by increasing node label. With this clear definition, for simple molecules with just a few bonds, it is possible to work out the serialization by hand.

## Results and discussion

### Validation and benchmarks

The program was validated using a shuffle test (also known as permutation test) with a manually curated test set of compounds, which are representative of the different domains of chemistry (organic and bioorganic compounds, organometal and coordination compounds, main group compounds, and clusters), as well as a library of highly symmetric “difficult graphs” which are more hypothetical than synthetically accessible, but are specifically designed to challenge the canonicalization algorithm [34, 35]. The largest tested molecule was an insulin derivative (CHEBI:5931) with 405 heavy (non-H) atoms and 416 bonds (edges). The whole “small” test set with 110 molecules was canonicalized and serialized in a few seconds on a laptop with 8 GB of RAM and an Intel(R) Core (TM) i7-6500U processor. In addition, a random selection of approx. 160,000 non-disordered and non-polymeric structures from the Cambridge Structural Database (CSD) was also subjected to the shuffle test which was passed without any issues.

### Comparison with other software

Many popular canonicalization procedures in chemistry are variants of the classical Morgan algorithm [21], which however uses the bond type of edges as an initial atom invariant and therefore is not domain-independent. Furthermore, non-equivalent atoms can still be assigned identical extended connectivity values, in particular in highly symmetrical molecules [23, 36], a problem that is particularly relevant to inorganic cluster chemistry. In contrast, TUCAN only relies on the atomic number as a chemistry-specific invariant. A major factor for string identifiers is the “character efficiency”. This was assessed for the largest compound in the current test, which is human insulin with a sum formula of C_257_H_383_N_65_O_77_S_6_. If one only considers the 405 heavy (non-H) atoms, the InChI string of the compound is 1586 characters long, while the TUCAN identifier requires 3158 characters and the canonical SMILES has 764 characters, according to PubChem [37]. Thus, a TUCAN identifier is about twice as long as an InChI string and four times as long as a canonical SMILES, which is mostly due to the “(”, “-”, and “)” characters used for representing each 2-tuple, while InChI and SMILES simply concatenate most of the symbols. However, arguably, most biological macromolecules are better represented by other encoding schemes anyway.

On the other hand, TUCAN is agnostic towards domain-specific concepts of structure and bonding, as it only considers the connectivity but not bond types. What is defined in the bond block of a molfile will be considered an edge in the internal graph representation and therefore it is fully up to the user to decide which atoms to connect. Furthermore, beyond the atomic number of each atom (node), the canonicalization is exclusively based on the topology of the molecular graph.

## Limitations

TUCAN is under constant development.^2^ Users should be aware of the following current limitations:

- The input molfile is assumed to be in valid v3000 format. The program will terminate when it encounters atom types which are not in line with the symbols recognized by IUPAC for elements 1 (H) to 118 (Og). In particular, the star (“*”) “pseudo-atom” is currently not processed and therefore, multi-center attachment is not allowed. Instead, all metal–ligand bonds have to be specified individually, which is particularly important for metallocenes.
- With the exception of the atom types, optional CHG, MASS, and RAD fields, and the connectivity of the molecule, no further information is read from the molfile. In particular, the current version ignores the xyz coordinates and differences in bond types. Most importantly, however, handling of stereochemistry will be the subject of a follow-up publication and therefore, diastereomers and enantiomers currently cannot be distinguished.
- The number of nodes (atoms) and edges (bonds) the program can handle depends on the underlying NetworkX package, which is limited only by the available RAM. The largest compound tested so far, human insulin with 405 heavy (non-H) atoms, did not pose a problem on a standard laptop. It has been suggested that graphs with more than 150.000 nodes can be handled by NetworkX.^3^ Lysozyme, for example, a 14.3 kDa protein, has a sum formula of C_613_H_959_N_193_O_185_S_10_ if one considers the eight cysteine residues to be fully reduced [38], which translates to 1001 heavy (non-H) and 959 hydrogen atoms or a total of 1960 nodes (= atoms). In addition, assuming “standard valences” for all atoms (C: 4, N: 3, O: 2, S: 2, H: 1), this gives an upper limit of approximately 4400 edges (= bonds) in the protein. In practice, however, the number of edges will be lower, as C=O double bonds, for example in the amide linkage of the peptide backbone, will internally be handled by the program as single edges. Therefore, it can be concluded that very likely even small- to medium-sized proteins can be canonicalized and serialized by the TUCAN in reasonable time, given that a proper molfile is available.
- Only atoms specified in the molfile are considered. Thus, “implicit hydrogens” are ignored and will not be added during graph generation. Canonicalization will still proceed on the “heavy atom core” but explicit addition of hydrogen atoms is strongly suggested. A “pre-processor” might be added to assist with the handling of “implicit hydrogens” on the carbon atom framework in future work.
- Tautomers are assigned different identifiers, but “hydrogen-pruned” versions of the molfile that only consider the heavy atoms will give identical TUCAN strings.
- Some string-based molecular representation such as SMILES [7] and SELFIES [39] have been explored for applications in artificial molecular design. Whether such generative models strictly require a “linear string” representation or can also work on the “tuple” notation proposed in the present work remains to be seen [40]. However, since the TUCAN string can be converted back to a molecular graph (or an adjacency matrix representation) without loss of information, it might serve as a link between string- and graph-based approaches for molecular generative models.

## Conclusion

TUCAN is a canonicalization and serialization tool that does not depend on domain-specific concepts of structure and bonding. This distinct feature will give molecular inorganic chemists an urgently needed tool to create identifiers for molecules that currently cannot be handled by InChI and SMILES. Beyond the atomic number, TUCAN relies exclusively on the molecular topology and therefore make it potentially applicable to non-chemical graphs as well. The serialization generates a unique “tuple-style” output which is fully bidirectional, allowing the TUCAN string to serve as both identifier and descriptor. Its utility in molecular generative models will be explored in future work. The current implementation does not distinguish between stereoisomers, but such an extension will be added in a future implementation. However, already in the present version, it is hoped that TUCAN will be useful for cheminformatics applications, in particular those dealing with molecules beyond the domain of organic chemistry.

## Data Availability

The program code as well as a curated test dataset in molfile v3000 format and “difficult” non-chemical graphs from Ref. [35] in dimacs format alongside additional program documentation is available on GitHub at https://github.com/TUCAN-nest/TUCAN.

## Abbreviations

ChEMBL: Chemical database of European Molecular Biology Laboratory (EMBL)
Cp: Cyclopentadienyl
InChI: International Chemical Identifier
IUPAC: International Union of Pure and Applied Chemistry
SMILES: Simplified Molecular Input Line Entry System
TUCAN: “Tuple canonicalization” or from Spanish “*tu canonicalización*”

## Footnotes

1 http://jmol.sourceforge.net/jscolors.

2 https://github.com/TUCAN-nest/TUCAN.

3 https://www.briggsby.com/large-internal-link-graphs-in-python#98m.
